# Individual Differences in Cognitive Coupling

**DOI:** 10.1093/geroni/igaf122.277

**Published:** 2025-12-31

**Authors:** Emorie Beck, Zoë Hawks, Eileen Graham

**Affiliations:** University of California, Davis, Davis, California, United States; Brooklyn Health, New York, New York, United States; Northwestern University, Evanston, Illinois, United States

## Abstract

Cognition can be understood in terms of both narrower cognitive domains (e.g., working memory, processing speed) and broader cognitive function (e.g., g, cognitive impairment) across the lifespan. Yet, there are at least two lingering challenges in our understanding of cognitive domains and global cognitive functioning. First, although cognitive abilities or capacities are relatively stable over time, cognitive function shows reliable and valid fluctuations in everyday life. Such fluctuations may provide insight into how modifiable risk factors of cognitive decline (e.g., exercise, cognitive activity, etc.) “get into the person” and increase their long-term risk for poor cognitive function. Second, current understandings of links between cognitive domains (i.e. the structure of cognitive functioning) is almost entirely between-person (i.e. people with good working memory also have fast processing speed), missing the possibility that cognitive domains may be differentially coupled within a person. Such within-person coupling across cognitive domains may be a key digital risk factor of long-term cognitive decline and dysfunction. Using an ecological momentary assessment of middle and older adults (N = 200, total obs.=12,431), we examine individual differences in the coupling of three cognitive domains (working memory, episodic memory, and processing speed) using continuous time structure equation models to capture both autoregressive (i.e. stability) and cross-lagged (i.e. directional cross-domain associations) among cognitive domains within- and between-persons. We will then examine whether individual differences in coupling are concurrently associated with global cognitive functioning, cognitive impairment, personality traits, and other psychosocial factors.

